# Microfluidic High-Throughput Platforms for Discovery of Novel Materials

**DOI:** 10.3390/nano10122514

**Published:** 2020-12-15

**Authors:** Peipei Zhou, Jinxu He, Lu Huang, Ziming Yu, Zhenning Su, Xuetao Shi, Jianhua Zhou

**Affiliations:** 1 Key Laboratory of Sensing Technology and Biomedical Instruments of Guangdong Province, School of Biomedical Engineering, Sun Yat-Sen University, Guangzhou 510006, China; peip.zhou@foxmail.com (P.Z.); hejx55@mail2.sysu.edu.cn (J.H.); Absurd1999@163.com (Z.Y.); yuzm@mail2.sysu.edu.cn (Z.S.); 2 School of Mechatronic Engineering, Guangdong Polytechnic Normal University, Guangzhou 510665, China; 3 National Engineering Research Centre for Tissue Restoration and Reconstruction, School of Material Science and Engineering, South China University of Technology, Guangzhou 510640, China; shxt@scut.edu.cn

**Keywords:** high-throughput platforms, materials screening, microarray, microfluidic droplet, micro/nano-structures

## Abstract

High-throughput screening is a potent technique to accelerate the discovery and development of new materials. By performing massive synthesis and characterization processes in parallel, it can rapidly discover materials with desired components, structures and functions. Among the various approaches for high-throughput screening, microfluidic platforms have attracted increasing attention. Compared with many current strategies that are generally based on robotic dispensers and automatic microplates, microfluidic platforms can significantly increase the throughput and reduce the consumption of reagents by several orders of magnitude. In this review, we first introduce current advances of the two types of microfluidic high-throughput platforms based on microarrays and microdroplets, respectively. Then the utilization of these platforms for screening different types of materials, including inorganic metals, metal alloys and organic polymers are described in detail. Finally, the challenges and opportunities in this promising field are critically discussed.

## 1. Introduction

The development of novel materials is of great importance to solve many industrial and social problems. However, seeking new materials and bringing them to industrial applications are usually time- and cost-consuming. Although the traditional method of “trial and error” is still the main approach to discover new materials, it cannot satisfy the increasing need for functional materials in current society. Therefore, it is of great urgency to develop high-throughput screening (HTS) that can reduce time and trial cycles for material discovery. HTS techniques are defined as approaches able to perform ten to a hundred thousand tests per day [1,2,3,4]. Additionally, the high-throughput platforms (HTPs) and high-throughput computational techniques are the most common methods of HTS techniques [5]. The capability of high-speed synthesis and analysis shows great potential to promote the development in material science, chemistry, pharmaceutical industry and biomedical engineering [6,7,8,9,10,11,12,13,14,15,16,17].

As an excellent example of high-efficiency experiments, HTPs can realize rapid synthesis, characterization and testing of numerous samples in a short period of time, screening out new materials with preferred performance. The approach of HTPs for material synthesis was pioneered over fifty years ago by Kennedy in 1965 [18], which allowed rapid and reliable screening of ternary-alloy isothermal sections. Subsequently, multiple-sample concept [19], parallel reactors [20] and combinatorial approach [21] were successively reported, and gradually applied for material production and screening [22,23,24,25]. To further overcome the disadvantages of high cost in time and price, great efforts were made to explore more effective and rapid approaches. Recently, microfluidic technology has become an attractive option owing to its superior properties, such as low consumption of reagents, excellent control of experimental conditions, high reaction efficiency, easy integration with online analysis, etc. [26,27,28,29,30,31,32,33].

In this review, recent advances in HTPs-based material discovery are discussed in detail. We start with an overview of two mainstream microfluidic screening strategies based on microarray and microfluidic droplets, respectively. Then, efforts focused on the applications of HTPs in discovering micro/nano-structured inorganic metals, metal alloys and polymers are reviewed, and several representative examples are highlighted. Finally, future challenges and opportunities in the promising research field are critically discussed.

## 2. High-Throughput Microfluidic Platforms

**Figure 1:**
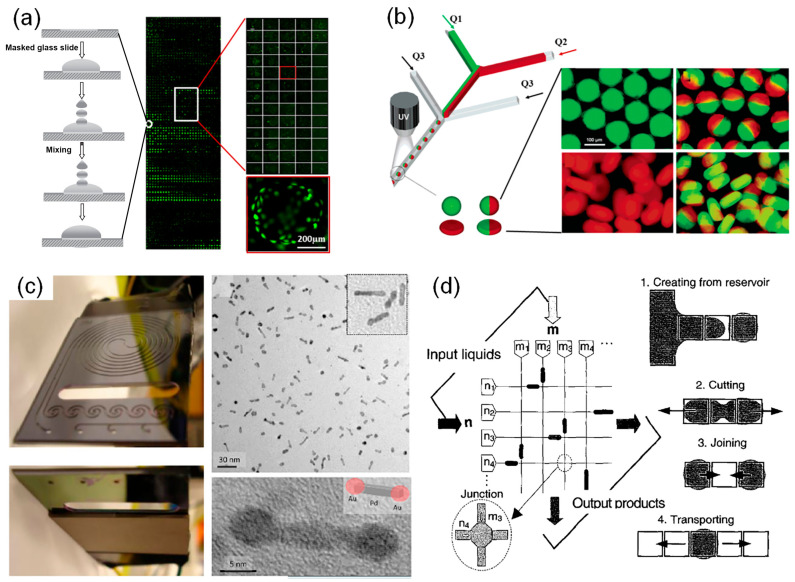
(**a**) A representative high-throughput platform (HTP) based on microarrays. A polymer hydrogel microarray with 2436 polymers (28 × 87 hydrogel spots) were prepared by inkjet printing. Subsequently, the microarray was incubated with HeLa cells for 48 h. Mosaic (the middle column in Figure 1a), and enlarged fluorescent images of cells were obtained. Reproduced with permission from [34]. Copyright Elsevier, 2009. (**b**) A representative HTP based on microdroplets. A microfluidic device was used to prepare colloid-filled hydrogel granules with different sizes and shapes by changing the flow rate of reagent 1 (Q1), reagent 2 (Q2) and oil (Q3). Reproduced with permission from [43]. Copyright American Chemical Society, 2006. (**c**) A representative continuous-flow microfluidic system was used to produce Au-Pd dumbbell nanoparticles. Reproduced with permission from [44]. Copyright American Chemical Society, 2017. (**d**) A digital microfluidic circuit and the four fundamental droplet operations: creating, cutting, joining and transporting. Reproduced with permission from [45]. Copyright IEEE Xplore, 2002.

Compared with traditional microplate-based HTPs that require samples of at least several microliters in each well, microfluidic platforms consume much less reagents with the scale of nanoliters to picoliters, which significantly reduces the cost and is beneficial to save rare samples. Microarray is one of the major microfluidic platforms, which integrates a large quantity of isolated reactors on one substrate. Additionally, each reactor is microscaled with volumes ranging from nanoliters to picoliters. It allows multiple parameters to be tested in parallel by simultaneously performing tens to thousands of experiments per batch. For example, Zhang and his coworkers developed a hydrogel microarray (Figure 1a), in which 2000 individual microgels with varying bioactivities were regularly patterned on a standard microscope slide, providing a high-throughput platform to rapidly screen desired polymers with thermal-responsive properties [34]. Perera et al. developed an automatic synthetic platform for drug discovery, which integrated commercially available components into a highly integrated module unit to perform both nanomole-scale reactions and micromole-scale syntheses [35]. This setup allows screenings of more than 1500 homogeneous reactions within 24 h under different temperature, pressure, and solvent, which has the advantages of real-time analysis, sufficient mixing, and avoidance of solvent evaporation. Due to the application of microarray-based HTPs, reactions were performed in parallel under a broad range of experimental parameters so that appropriate conditions for generating nanostructures with specific morphologies can be rapidly identified. Moreover, Duffy et al. described a hydrogel microarray that integrated 80 unique holes on a single microscope slide using three-dimensional (3D) printing [36]. By filling the holes with double network hydrogels, the novel platform offered a powerful tool to screen hydrogels with desired compressive and tensile properties, which could be further optimized for drug delivery, cell encapsulation, and tissue engineering. Microarrays have also been widely applied in a wide range of biomedical applications, such as pharmaceutical discovery, small molecule and protein screening, toxicity tests, etc. [37,38,39,40,41,42]. For example, Hay et al. used the polymer microarray with high content screening system and Pathfinder software to screen and discover new extracellular substrates, which can promote hepatic endoderm, drug-inducible metabolism and toxicology [38]. Additionally, Khan et al. proposed a microarray platform combined with a high-throughput screening approach to screen and analyze the biological functionality of 135 polymer blends, leading to the identification of cell-compatible biopolymers permissive for human skeletal stem cell growth in both in vitro and in vivo applications [40].

Despite the improvement in throughput, microarray-based HTPs are still limited in many cases that required higher screening efficiency. To address the issue, microdroplet technology has drawn increasing attention and been developed for high-throughput screenings [46]. Microfluidic droplet chips can be divided into continuous microfluidic chips (Figure 1b,c) [47,48,49,50,51] and digital microfluidic chips (Figure 1d) [52,53,54]. Shepherd’s group provided a continuous microfluidic device (Figure 1b) to generate monodisperse colloid-filled hydrogel particles with different shapes and compositions [43]. Additionally, Jensen et al. described a new device for the production of Au-Pd dumbbell-like nanostructure with high electrocatalytic activity [44]. This device was integrated with a sequential-addition microfluidic reactor and an ultrasonic to control the growth of Au onto the both sides of Pd nanorods (Figure 1c). As the key platform of microdroplet technology, continuous microfluidic chip can generate monodisperse droplets (usually at nano- or picoliters) at very high frequencies (from tens to thousands of droplets per second) [55,56]. Additionally, each microdroplet serves as an independent microreactor, in which synthesis of materials can be carried out without interference under certain conditions. Digital microfluidics employed electrowetting to control and discretize the continuous flow into individual droplets. Sung et al. fully reported the functional digital microfluidic circuits and the four fundamental droplet operations mechanisms [45]. It provides a promising experimental platform with advantages of a fast response, high precision, and digital readouts. Microdroplet-based HTPs has many advantages [57,58,59]. Firstly, it consumes much less reactants since the working volume of a plate well (e.g., 10 μL for each well of a 384-well plate) is ten million times that of a single droplet (1.0 pL) [60]. Secondly, the high surface-to-volume ratio of microdroplets and short diffusion distance in microdroplets result in pronounced acceleration of reactions and thus can significantly shorten the screening time. Thirdly, it provides chemical and physical confinement to avoid cross-contamination. Using this technique, a large quantity of independent experiments can be easily performed within a very short period and only a small amount of reagents are consumed.

## 3. Current Applications of HTPs for Material Synthesis

To date, HTPs have been extensively applied to discover novel materials including metal nanoparticles, metal alloy nanoparticles, quantum dots, organic nanoparticles, combinational polymers, metal-organic frameworks, perovskites and so on, which show promising applications in biosensing, catalysis, energy storage and drug delivery. Herein, a comparison among the various HTPs is presented in Table 1, including their types, platform materials, reactants, the screening materials and advantages. Following the table, a few examples of HTPs-based material screening reported in recent years are highlighted, which are ordered by inorganic metals, inorganic metal alloys, inorganic biomaterials and organic polymers.

### 3.1. Inorganic Metals and Metal Alloys

**Figure 2:**
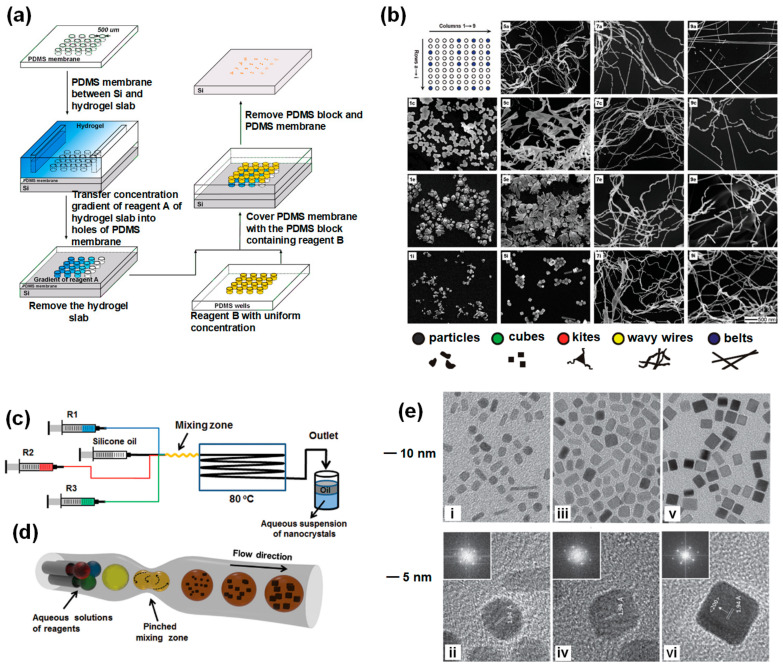
(**a**) Schematic of the fabrication process of a high-throughput array reactor for screening Au nanostructures. The synthesis of Au nanostructures involves the mixing of the HAuCl_4_ solution with cetyltrimethylammonium bromide (CTAB) solution. Then, addition of the L-ascorbic acid solution reduces Au to Au(I), and addition of NaOH at Au(I) state boosts the reducing power of L-ascorbic acid to further reduce Au(I) to elemental Au. (**b**) Scanning electron microscopy (SEM) images of Au nanostructures that were formed in a two-dimensional array of reactors with gradients for the concentrations of NaOH and CTAB. Reproduced with permission from [61]. Copyright John Wiley and Sons, 2011. (**c**) High-throughput droplet platform integrated with the mixing tube (**d**) for screening the synthesis of Pd. The preparation of Pd nanocrystals involves the reduction of Na_2_PdCl_4_ by L-ascorbic acid in an aqueous solution at 80 °C, in the presence of KBr and poly(vinyl pyrrolidone) (PVP); (**e**) Transmission electron microscope (TEM) (i,iii,v) and high resolution transmission electron microscope (HRTEM) images (ii,iv,vi) of Pd nanocrystals with different morphologies. Reproduced with permission from [84]. Copyright John Wiley and Sons, 2013.

High-throughput screening of metal materials is one of the attractive applications of HTPs. As illustrated in Figure 2a, Zhou et al. described a simple microarray reactor with one- or two-dimensional gradients, which can quickly screen the synthetic conditions for metal nanostructures with desired morphologies [61]. In this approach, concentration gradients of four reagents were established on one polydimethylsiloxane (PDMS) block containing an array of microwells. By using the concentration gradients, metal nanoparticles prepared under 9 × 9 types of experimental conditions were screened at the same time (Figure 2b). Utilizing the platform, metal nanostructures including Au and Pd with various morphologies could be generated under different reagent concentrations, pH values and temperature in one experiment. Additionally, the desired nanostructures and their synthetic parameters could be rapidly obtained. In addition, an array microreactor has been developed to screen a Pt-Pd-In ternary library of 66 compositions for the desired catalytic properties [83]. It was also applied to identify the dehydrogenation of cyclohexane to benzene.

In addition to the microarray, microdroplet-based HTPs are also utilized for the syntheses and selection of inorganic metals. Due to the flexible controllability in reaction stoichiometric ratio, reaction time, temperature and other experimental parameters, HTPs based on microfluidic droplets have been extensively applied in the preparation of micro-/nano-size metals and metal alloys [85,86,87,88,89]. For example, Kim et al. demonstrated a simple droplet-based microreactor to generate Pd nanocrystals with controlled shapes and sizes (Figure 2c) [84]. The microfluidic platform was produced by commercial polytetrafluoroethylene (PTEE) tubes and silica capillaries that are cost-effective. Additionally, a periodically pinched segmentation was introduced to improve the efficiency of the mixer (Figure 2d). By adjusting the concentrations of L-ascorbic acid, different morphologies of Pd nanocrystals were obtained. As the amount of L-ascorbic acid increased, the Pd nanocrystals with round shape turned into nanobars with sharp corners (Figure 2e). The dimensions of Pd nanocrystals were also tuned by adjusting capping agents. This simple and cost-efficient setup provided a way to obtain nanocrystals with well-controlled sizes and shapes by screening varying reaction conditions. Jensen et al. have successfully synthesized and characterized Au-Pd dumbbell nanoparticles based on a continuous-flow microfluidic system [44]. It was integrated with a sequential-addition microfluidic chip and an ultrasonic field. Additionally, the obtained Au-Pd dumbbell nanoparticles showed better electrocatalytic performance than pure Pa particles. Additionally, Kyoung et al. proposed a droplet-based microfluidic device that employed polymeric hydrogel and cell extracts to establish artificial cell bioreactors, in which in vitro biosynthesis of Fe, Au and other metal nanoparticles were achieved [90]. In this bioreactor, two aqueous phases with different reagents were flowed through the orifice of the microchannel, and subsequently massive droplets were rapidly generated by shear-off force from the oil phase flow. The on-chip microdroplet-based cellular bioreactor offered an efficient platform to synthesize and screen metal nanoparticles with high biocompatibility and bioactivity, and could help to reveal the mechanisms of cellular detoxification. Additionally, Zhang and his coworkers proposed a counter-flow mixer in a microfluidic droplet chip to effectively accelerate the mixing process of solutions for the synthesis of Au, Pd and Pd-M with different sizes [91]. This setup was different with regular microfluidic devices, which integrated multiple functions including reaction, cooling, water and oil separation and purification. Additionally, this setup can serve as a simple, scalable and cost-effective high-throughput platform to produce uniform and well-controlled metal nanoparticles.

In recent years, the alloy quantum dots have also been widely concerned, such as CdSeTe [92,93], ZnSe/ZnS [94], etc. Doping new elements into alloy quantum dots would adjust their luminescence characteristics and emission wavelengths, and widely improve their quantum yield as well. Since the fascinating electronic and photonic properties of the alloy quantum dots are highly dependent on size and shape of quantum dots, which are affected by the pyrolysis process during the production, it is necessary to accurately control and screen the reaction parameters of pyrolysis. Yao et al. developed a microfluidic droplet reactor to prepare a series of different colored fluorescent CdTe quantum dots by precisely control the temperature and the time of crystal growth [95]. Furthermore, Chen et al. used a microarray of 3 × 3 with the Taguchi method to screen the performance of the Li_2_SrSiO_4_ phosphor under different concentrations of Eu^2+^, Ce^3+^ by evaluating the luminescence efficiency, color rendering index and color temperature [96].

### 3.2. Inorganic Biomaterials and Organic Polymer

**Figure 3:**
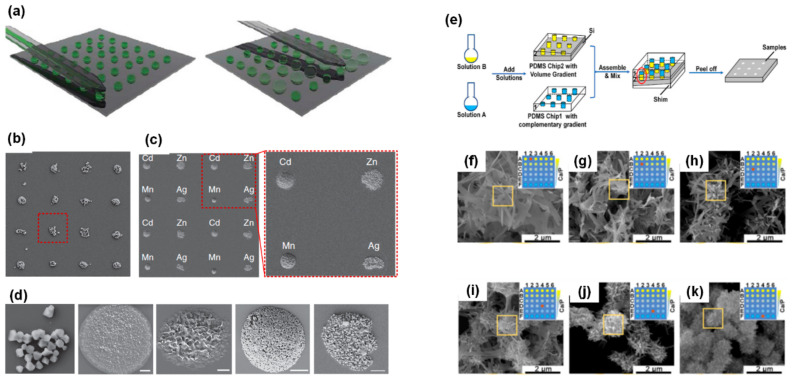
(**a**) Schematic illustration of two-step microfluidic pen lithography method, showing an array with precise delivery of different volume of a solution. (**b**) The synthesized HKUST-1 array. (**c**) The 4 × 4 array of the M-Prussian blue analogues, where M is Cd(II), Zn(II), Mn(II) and Ag(I). (**d**) The SEM images of HKUST-1, Cd-PBA, Zn-PBA, Mn-PBA and Ag-PBA nanocrystals (from the left images to the right, and scale bars are 2 μm). Reproduced with permission from [107]. Copyright Nature Communications, 2013. (**e**) Schematic illustration of fabricating reactors based on microarrays, showing an array with precise partially perforated holes used to deliver solutions. (**f**–**k**) SEM images of calcium phosphates structures for screening the experimental concentration ratio of Ca(NO_3_)_2_ and (NH_4_)_2_HPO_4_ (scale bars are 2 μm), (**f**) C_ca_/C_p_ = 2.5/0.5; (**g**) C_ca_/C_p_ = 2.1/0.9; (**h**) C_ca_/C_p_ = 1.7/1.3; (**i**) C_ca_/C_p_ = 1.3/1.7 and (**j**) C_ca_/C_p_ = 0.9/1.3; (**k**) C_ca_/C_p_ = 0.5/2.5. Reproduced with permission from [108]. Copyright Elsevier, 2020.

Apart from the screening of inorganic materials, HTPs have also been increasingly employed for high-throughput screening of organic compounds, which show various applications in biosensing, drug and gene delivery [97,98,99,100,101]. For instance, the screening tests of metal-organic frameworks (MOFs) [102,103,104,105,106] have drawn immense attention due to their diverse structural topologies and tunable chemical functionalities. Additionally, the conventional tests may take several hours or days for MOFs synthesis with costly microdevices. In order to overcome these barriers, Carlos’s group developed the technique of microfluidic pen lithography (MPL), which could create mixed femtolitre droplet arrays using microfluidic pens (MPs) [107]. The working principles of MPL contained two steps (Figure 3a). Firstly, an array of droplets containing the first type of solution was prepatterned by MPs (step 1). Then, the second type of solution was delivered to the patterned area to mix and react with the first one (step 2). The results showed that MPL enabled the independent synthesis of MOFs at every spot and successfully created a multiplexed MOFs array (Figure 3b–d). This flexible technique is also promising for high-throughput screening and discovering of other novel materials. Additionally, in principle, it can realize syntheses of ten thousand samples of MOFs by MPL. Li et al. have designed a microarray platform, which can rapidly screen the experimental conditions for producing calcium phosphates (CaP), as shown in Figure 3e [108]. CaP was prepared by mixing a Ca(NO_3_)_2_ solution with an (NH_4_)_2_HPO_4_ solution. In this technique, the gradients of concentration ratio of Ca/P and NaOH concentration were achieved by applying microarray holes with different heights. Figure 3f–k show the SEM images of CaP synthesized under different concentration ratios (Ca/P) between two vital reactants of calcium nitrate tetrahydrate (Ca(NO_3_)_2_) and ammonium phosphate dibasic [(NH_4_)_2_HPO_4_]. In their platform, the experimental conditions of reaction concentration and pH values were manipulated, microparticles quickly screened and CaP micro/nanostructures with diverse morphologies were synthesized under particular conditions. Additionally, this technique was universal, which therefore was promising to be applied to other materials. In addition, Hook et al. also developed a high-throughput microarray to screen thermo-responsive polymers by measuring water contact angle (WCA) [39]. The WCA of each polymer was acquired by the circle-fitting method [109]. Additionally, the time-of-fight secondary ion mass spectrometry (ToF-SIMS) with surface sensitivity and molecular specificity was adopted to study the surface enrichment of the molecular fragments under different temperatures. This microarray was successfully used to identify 279 unique polymers with thermo-responsive properties as the temperature was switched from 8 to 40 °C.

**Figure 4:**
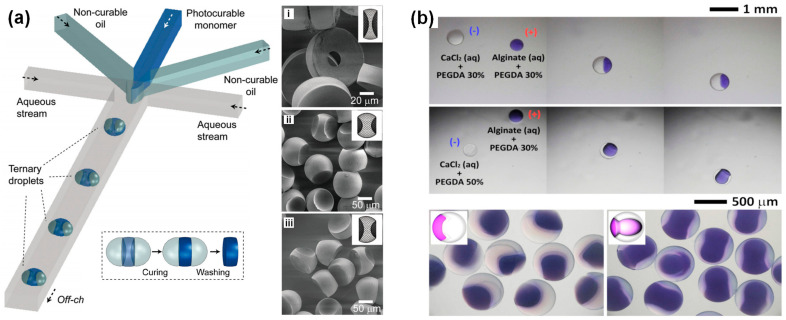
(**a**) On-chip synthesis of biconcave polymer microparticles with ternary components. Additionally, SEM images of biconcave polymer microparticles with different structures, which were produced from droplets by changing the flow-rate ratios of the droplet phases. Reproduced with permission from [110]. Copyright John Wiley and Sons, 2015. (**b**) Fabrication of Janus microparticles with different shaped via electric-field-induced droplet dispensing into oil based on digital microfluidics. Reproduced with permission from [111]. Copyright Nature, 2016.

The microdroplet-based HTPs also make contributions to search polymeric materials with unique micro/nanostructures. For example, Nisisako [110] utilized a ternary droplet structure to produce various types of polymer particles, as shown in Figure 4a. A light-sensitive and two light-insensitive fluids were introduced from three separated inlets as the inner phase. Then, the inner phase composed by multifluids was dispersed by the continuous phase (i.e., aqueous stream) to generate the ternary droplets. Lastly, the produced ternary droplets were prepared in the cylindrical microcapillary and polymerized by ultraviolet light to obtain spherical and homogeneous concave particles. Leveraging the capabilities of generating uniform and well-controlled biconcave particles, this device can also be applied to synthesize functional microelements with concave structures for targeted drug delivery and other applications. Um et al. [111] proposed an integrated platform to prepare colloids and Janus microparticles with different structures (Figure 4i). The platform firstly used conventional nozzles to dispense charged droplets into oil. Then, the positively charged droplets and the negatively charged ones were merged by electric attraction. Afterwards, the mixed droplets were polymerized by UV light to produce Janus droplets with anisotropic or isotropic structures. The structures can be controlled by the concentration. This platform provided a useful and flexible technique to manipulate microparticle synthesis. Moreover, a double-emulsion microfluidic chip composed of two connected droplet forming stages was also designed and manufactured to produce particles with different morphologies [112]. The water–gel microparticles with shapes of meniscus or multipods were steadily prepared under the synergistic effect of geometric restriction and the inhibition of interfacial polymerization reaction. Based on the chip, particles with various novel shapes could be produced with a higher degree of flexibility.

## 4. Conclusions and Prospective

In this review, we focused on recent advancements of microfluidic HTPs for searching materials with novel micro/nanostructures. Firstly, two major formats of microfluidic HTPs based on microarrays and microdroplets are described, respectively. Then the widespread utilizations of HTPs in discovering inorganic and organic materials with desired micro/nanostructures are comprehensively compared and summarized. Furthermore, a few examples of HTPs-based material screenings developed in recent years are discussed in detail.

**Figure 5:**
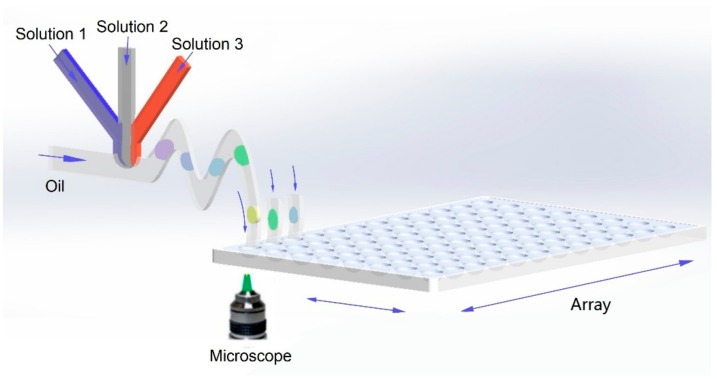
A demonstration of high-throughput-screening platform that combine droplet generator, microarray and high-throughput characterization for screening material structures.

Both microarrays- and microdroplets-based HTPs can significantly increase the screening throughput and accelerate development of material science. For microarray-based HTPs, the synthetic parameters of each reaction can be precisely encoded by spatial coordinates, but the throughput is limited by device area and density of reaction sites. For microdroplet-based HTPs, the throughput greatly increases owing to the continuous and rapid generation of microdroplets. However, it remains challenging to accurately encode the synthetic parameters of each microdroplet, thus limiting the further increasement of its throughput. Recently, a novel high-throughput method called “droplet library”, which combines a microfluidic droplet generator with microarrays, are proposed [75,113]. The basic principles are shown in Figure 5. Firstly, droplets containing small compounds are prepared by parallel microfluidic devices and subsequently transported to microarray plates. Then the following droplets with different compounds could be gathered in one tube as a droplet library. The droplet library was then reinjected into another device to mix with a target for screening the compounds with optimal antimicrobial activities. This integrated platform takes significantly less time than conventional microdroplet-based HTPs. Although mainly applied for biological experiments, such as investigations of antimicrobial activities, pharmacological screening, drug-resistance analysis, etc., the novel integrated approach shows great potentials in screening materials with ultra-high throughput, providing a promising approach towards the development of next-generation HTPs.

Moreover, to achieve truly high-throughput screening, it is necessary to establish highly integrated HTPs with multiple functions of material synthesis, characterization and data analysis. Zhou et al. [114] have proposed a high-throughput screening system. It combined a microfluidic reactor to generate hydrogel droplets with different crystals of drugs, a camera to capture the optical images of the droplets, and deep learning to analyze and classify the obtained images. Additionally, the microfluidic chip was fabricated with a flow-focusing geometry to produce droplets. Their system offered a new high-throughput platform and could be applied to quickly synthesize the massive materials and accurately analyze the data. With massive materials informatics and databases, it offers a potent platform to accelerate the development of the new materials. Despite the great advancements in material synthesis, the performance of current HTPs in high-throughput characterization is still far from satisfactory. Therefore, developing compatible high-throughput characterization techniques to combine with synthetic modules is one of the important trends of future HTPs. Additionally, as HTPs usually produce massive data, approaches for high-throughput data processing are also in great demand. Machine learning is a powerful tool to process and analyze massive information, which shows promising applications in future HTPs. Since the application of HTPs has gradually played a critical role in new material preparation, it will show significant impact on the development of material science, biological science, biomedical engineering and military science in the future.

## Figures and Tables

**Table 1:** Examples of high-throughput platforms and their advantages in materials screening.

Types of HTS Platforms	Platform Materials	Reactants	The Screening Materials	Advantages and Applications	Year	Ref.
A simple microarray reactor with one- or two-dimensional gradients	PDMS microarray, 9 × 9 micro-pores	HAuCl_4_ solution + cetyltrimethylammonium bromide (CTAB) solution + NaOH solution	Au, Pd	The morphologies of metal nanostructures under different experimental conditions	2011	[61]
A microarray of polymer hydrogel	Microscope slide	Hydrogel + cells	Smart polymer with desired properties	Cell encapsulation	2009	[34]
A high-throughput microarray with ToF-SIMS	279 materials spots of two-generation microarray	Polymer materials spots	279 unique materials with thermo-responsive properties	Discovery of novel switchable materials, and development of new way for high-throughput characterization	2013	[39]
A patterned superhydrophobic platform with hydrophilic spots	Microarray based on chitosan and alginate	Osteoblast-like, fibroblasts, the scaffolds modified with fibronectin	The most favorable materials for cells	Discovery of the most favorable conditions for the culture of each cell type, and rapid collection of reliable and valid data	2013	[62]
High-throughput array of cells and biomaterials via laser printing	Sodium alginate	Nano-HA + cells + sodium alginate solution	HA nanocrystals, 577 patterns with EA. hy926 cells after live/dead	Biopolymers, nano-sized particles of HA, human endothelial cells and 3D biostructures	2010	[63]
Combinatorial polymer microarray	pHEMA and glass slide	Homopolymers + 3 different green fluorescent proteins (GFPs)-labeled bacterial species	Biomaterials with unique (meth)acrylate monomers	Discovery of novel materials with broad resistance to bacterial attachment	2013	[64]
Microfluidic platform of ultra-small gold	Thermoplastics (PE and PEEK)	Mercaptobenzoic acid/CTAB + HAuCl_4_ + NaBH_4_ + AgNO_3_ + ascorbic acid	Au (spheres, 2–40 nm) and Au (nanorods, 10 nm × 50–100 nm)	Biosensing (chemical sensing, plasmonic functionalities, proof-of-concept)	2013	[65]
					2015	[66]
					2016	[67]
A millimetric coaxial microfluidic device	PDMS	FeCl_3_ + FeCl_2_ + TMAOH	Fe_3_O_4_ (spheres, <7 nm)	Open the way to other experiments, MRI imaging	2008	[68]
A microfluidic platform using two microreactors operating under different temperature and flow continuous	PDMS	FeCl_3_ + FeCl_2_ + HCl + TMAOH	Goethite	Promoting a rapid homogeneity of reactants, MRI imaging	2009	[69]
One-step synthetic microreactor based on continuous droplets	Glass	FeCl_3_ + FeCl_2_ + HCl + ZnCl_2_ + NH_4_OH	Zn doped Fe_3_O_4_ nanoparticle with different sizes	Allowing greater control on the chemical stoichiometry, Fluorescence imaging	2015	[70]
Microfluidic chips using a staggered herringbone micromixer	PDMS and glass slide	PCDA + DMSO + DI water	The fluorescence signal of PDA under different sizes	Stimulus-responsive fluorescence, improving the production for	2016	[71]
Microfluidic chips with different junction reactor	Alloy (stainless steel) Glass PDMS	CFA + acetone + isopropyl ether; PLGA + HPCS + AcDX + PTX PLGA-PEG + CAN + H_2_O	Polymeric with different size (spheres)	drug delivery	2010	[72]
					2015	[73]
					2008	[74]
Multi-microfluidic platforms for high-throughput production of nanoparticles	PDMS	Ad-PEG + Ad-PEG-RGD + As-PEG-TAT + CD-PEI + BSA-Cy5 + HRP-RhB + pEGFP	Colloidal nanocrystals/TFs	Immunotherapy, stem cell reprogramming	2016	[75]
Gas-liquid multi-phase microfluidic droplet platform for shape-controlled continuous synthesis	Spiral silicon/pyrex	Oxygen + Pd precursor + ethylene glycol + bromide ions	Pd with different nanostructures	Catalysis, molecular detection and biomedical Phototherapies	2016	[76]
A microfluidic reactor with segmented flow	Spiral	Na_2_PdCl_4_ + KBr + H_2_O + EG + PVP + Air	Pd nanorods	High activity catalytic hydrogenation of styrene	2016	[77]
A microfluidic chip with photoinitiated polymerization	PDMS	Hydrogel PEGDA + PEG + PI	Photopolymerized hydrogels encapsulated API crystals	Drug delivery	2019	[78]
Digital microfluidic high-throughput printing	Plates, ITO coated glass and hydrophobic Teflon-AF Layer	Cu (II) dimers and 1,3,5-benzenetricarboxylate	HKUST-1 crystals	Huge production of MOF crystals with different functionalities	2012	[79]
Digital microfluidics	Glass substrates, copper wire	CsPbBr_3_ NCs and a Hyflon AD 60 fluoropolymer	CsPbBr_3_ NC-Hyflon films	Temperature sensor	2020	[80]
An electrowetting-on-dielectric digital microfluidic platforms	A glass wafer and an indium tin oxide layer	Menthol+Triethylamine + 4-(dimethylamino)pyridine + acetic anhydride	Engine-and-cargo droplets with different shapes	Kinetics study, solvent screening, catalyst loading optimization	2019	[81]
A reaction platform based on digital microfluidics	Quartz glass, polylactic and copper wire	FeCl_3_·6H_2_O + FeCl_2_·4H_2_O + NaOH + PFOTES + Silica	Superparamagnetic hydrophobic particles	Bio-chemical analysis	2016	[82]
